# Performance and Micro-Pacing Strategies in a Freestyle Cross-Country Skiing Distance Race

**DOI:** 10.3389/fspor.2022.834474

**Published:** 2022-02-17

**Authors:** Craig A. Staunton, Steffi L. Colyer, Øyvind Karlsson, Mikael Swarén, Simo Ihalainen, Kerry McGawley

**Affiliations:** ^1^Swedish Winter Sports Research Centre, Department of Health Sciences, Mid Sweden University, Östersund, Sweden; ^2^Department for Health, University of Bath, Bath, United Kingdom; ^3^Swedish Unit for Metrology in Sports, School of Education, Health and Social Studies, Dalarna University, Falun, Sweden; ^4^Research Institute for Olympic Sports, Jyväskylä, Finland

**Keywords:** GNSS, GPS, skate skiing, statistical parametric mapping, tactics

## Abstract

This study examined the micro-pacing strategies during a distance freestyle cross-country (XC) skiing competition. Nine female and 10 male highly trained XC skiers wore a GNSS device during a FIS-sanctioned race. The course was ~4900 m; women completed two-laps; men completed three-laps. The course was divided into uphill (S1, S3, S5, S7), downhill (S2, S4, S6, S8), and flat (S9) sections for analyses. Statistical parametric mapping was used to determine the course positions (clusters) where total race time or section time was significantly associated with instantaneous skiing speed. Total race time was associated with instantaneous skiing speed during a cluster in S1 on lap 2 for both sexes (t ≥ 5.899, *p* ≤ 0.008). The two longest uphill sections (S1; S5) and the flat section (S9) contained clusters where section times were related to instantaneous skiing speed for both sexes (*p* < 0.05). The fastest woman gained 6.9 s on the slowest woman during a cluster in S1 on lap 1 and 7.3 s during a cluster in S9 on lap 1. The fastest man gained 51.7 s on the slowest man over all clusters in S5 over the 3 laps combined. Compared to skiers with longer total race times, skiers with shorter race times skied with faster instantaneous speeds in some clusters of the uphill sections, as well as on the flat section of the course. This study also identified different relative micro-pacing strategies for women and men during freestyle distance XC skiing races. Finally, statistical parametric mapping analyses can help to identify individual strengths and weaknesses for guiding training programs and optimise competition pacing strategies.

## Introduction

International Ski Federation (FIS) cross-country (XC) skiing competitions are typically categorised into two main formats for individual events: sprint skiing (1.0–1.8 km) and distance skiing (≥ 10 and ≥ 15 km for women and men, respectively), and use either the freestyle or classical technique (The International Ski Federation, 2020). In addition to various competition distances and techniques, a further distinguishing characteristic of XC skiing events is the undulating terrain. The topography varies throughout the courses and contains approximately equal distances of uphill, downhill and flat sections (The International Ski Federation, 2020). Each of these factors influence a skier's distribution of effort throughout skiing races (i.e., the skier's pacing strategy) (Stöggl et al., 2018).

Researchers have previously focused on lap-to-lap pacing strategies adopted by XC skiers via changes in lap times over the duration of a race (Stöggl et al., 2018). More recently, it has been observed that skiers apply specific micro-pacing strategies within laps, which might be related to variations in the course topography (Andersson et al., 2010; Sandbakk et al., 2011, 2016; Ihalainen et al., 2020). For example, uphill sections have been identified as particularly critical to successful performance (Andersson et al., 2010; Sandbakk et al., 2011, 2016). In these sections, XC skiers typically exercise at intensities above their maximal aerobic power in order to maintain speed (Karlsson et al., 2018; Gløersen et al., 2020). Further, Ihalainen et al. (2020) observed that instantaneous skiing speed during distinct course sections, measured by a global navigation satellite system (GNSS) device, was related to overall sprint skiing performance in female skiers. Specifically, this study (Ihalainen et al., 2020) used a novel statistical technique termed statistical parametric mapping (SPM), which has recently been promoted as a useful tool for statistically analysing smooth continuous biomechanical data (Pataky, 2010, 2012). The use of GNSS and SPM can enable the comparison of skiing speeds between athletes at standardised course locations. The results from Ihalainen et al. (2020) identified that the skiing speeds during transitions between uphill and flat sections, and from flat or uphill to downhill sections, were strongly related to shorter race times (i.e., better performance).

It has been suggested that pacing strategies in XC skiing might vary depending on sex (Losnegard et al., 2016; Andersson et al., 2019; Ardigò et al., 2020), skiing technique (Stöggl et al., 2018; Ardigò et al., 2020) and competition distance (Losnegard et al., 2016). For instance, men tend to ski with a greater relative power output during uphill sections compared to women (Andersson et al., 2019). Additionally, men tend to ski faster particularly during transition periods between flat and uphill sections compared to women (Ardigò et al., 2020). Further, Losnegard et al. (2016) observed that both women and men adopted positive lap-to-lap pacing strategies during skating and classical technique competitions (i.e., faster initial laps compared to later laps). However, the fastest male skiers were able to maintain their lap speeds to a greater extent than their slower counterparts, whereas female skiers exhibited similar reductions in speed despite different overall performance levels. In addition, greater reductions in skiing speed were observed from the first to the last lap of a distance race with the classical technique compared to the skating technique (Losnegard et al., 2016). Finally, previous studies have highlighted that the reductions in skiing speed between laps appear to be greater in sprint compared to distance skiing (Stöggl et al., 2018). All taken together, it appears that sex, skiing technique and competition distance might influence pacing strategies in XC skiing.

Previous investigations of pacing strategies in XC skiing have focused mainly on sprint skiing (Andersson et al., 2010, 2016; Sandbakk et al., 2010, 2011), while less attention has been given to distance races (Stöggl et al., 2020). Stöggl et al. (2020) identified that elite-level skiers are capable of maintaining a more even pacing strategy during a long-distance skiing race, whereas amateur skiers tend to adopt a more positive pacing strategy. However, this study analysed only mean skiing speed through different sections of a course, rather than identifying how instantaneous speeds relate to section or total race times (Stöggl et al., 2020). To date, no study has applied SPM analyses to continuous GNSS data to understand within-lap micro-pacing strategies during distance skiing. Accordingly, it remains unclear how within-lap micro-pacing strategies relate to race performance during distance competitions for both women and men using the freestyle technique. Therefore, the aim of the present study was to apply SPM analyses to continuous GNSS data to analyse the within-lap micro-pacing strategies during a women's and men's distance freestyle XC skiing competition. This information could guide coaches and skiers in identifying how best to optimise pacing strategies and improve performance during these events.

## Materials and Methods

### Participants

Nine female (age: 24 ± 3 years; distance FIS points: 73 ± 13) and ten male (age: 22 ± 1 years; distance FIS points: 68 ± 22) tier 3 athletes (McKay et al., 2022) competing in FIS-sanctioned freestyle distance XC skiing races agreed to participate in this study. All skiers provided written informed consent prior to participation and subsequently completed all requirements of the study. Ethical approval was granted by the ethical review board of Umeå University, Sweden (registration number: #2018-441-32M). All research was conducted in accordance with the Code of Ethics of the World Medical Association (Declaration of Helsinki).

### Design

Skiers wore a commercial GNSS sensor (Catapult OptimEye S5, Catapult Sports, Melbourne Australia; dimensions: 96 × 52 × 13 mm; mass: 67 g) throughout the duration of the individual time-trial XC skiing races. The GNSS sensor was positioned on the centre of the skier's upper back at approximately the level of the superior angle of the scapulae using the harness provided by the manufacturer. The GNSS sensor recorded the skier's position at a sample frequency of 10 Hz throughout the duration of the race.

**Figure 1:**
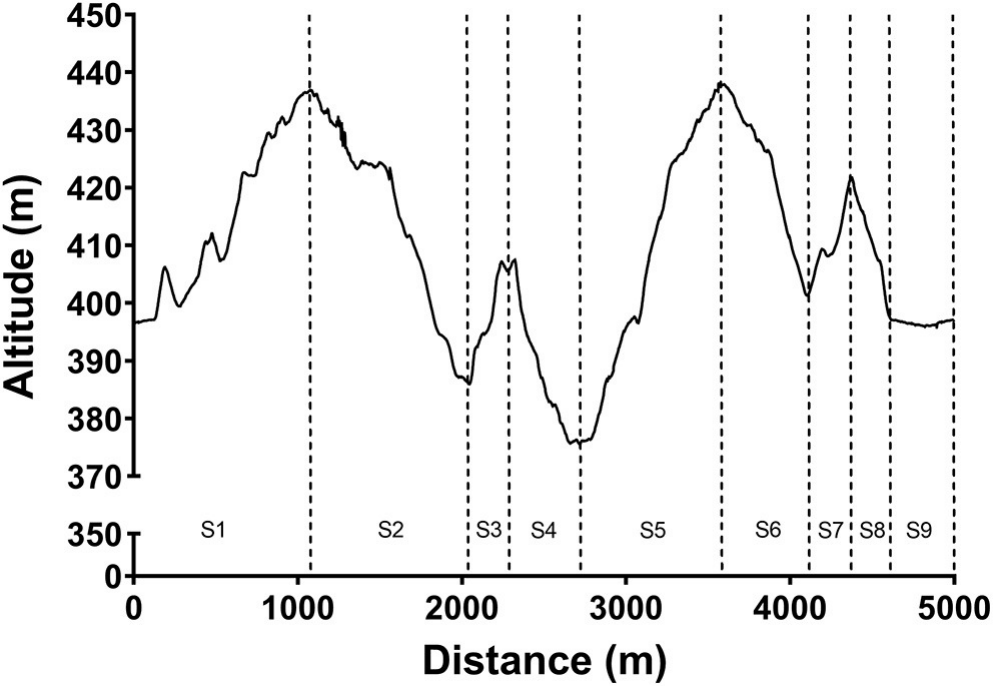
A schematic of the course profile including specific uphill (S1, S3, S5, S7), downhill (S2, S4, S6, S8), and flat (S9) sections.

Prior to commencement of the race, skiers completed their usual warm-up and preparation routines. All preparation of the skis was conducted by professional ski technicians. The competition course was ~4,900 m in distance, with a maximum climb of 63 and 165 m of total climbing. The women competed over two laps (total distance: 9,743 m) and the men competed over three laps (total distance: 14,678 m). As shown in Figure 1, the course was divided into discrete uphill (S1, S3, S5, S7), downhill (S2, S4, S6, S8), and flat sections (S9) for subsequent analysis. Due to different positions of the start, split and finish lines, the lap and section lengths varied slightly. For example, S1 on the second and third laps were slightly shorter (by ~100 m) compared to the first lap. In addition, on the final lap for both the women and men, S9 was not included in the analyses since the finish line was close to the bottom of the final downhill section (S8).

### Data Analysis

In order to apply a coordinate mapping procedure to the GNSS race data, a mapping trajectory was measured before the competition start using a differential GNSS (dGNSS) device (Leica Zeno GG04+, Leica Geosystems, Switzerland). The dGNSS device had a 1 cm ± 1 ppm horizontal and 2 cm ± 1 ppm vertical positional accuracy in the real-time kinematics mode that was used. Subsequently, positioning data from the race GNSS device was corrected using a reference trajectory according to methods previously described (Gløersen et al., 2018a; Ihalainen et al., 2020). Briefly, the positioning coordinates of the reference trajectory were filtered using a second order low-pass Butterworth filter set at 0.3 Hz. The filtered coordinates were subsequently resampled to every 1-m interval along the course using cubic spline interpolation. The GNSS positioning coordinates were then filtered using the same Butterworth filter. Filtered GNSS coordinates were subsequently mapped onto the reference trajectory, where the Euclidean distance between the measured position and the reference trajectory was minimised. The corrected GNSS data was then linearly interpolated to every 1-m interval to permit comparison of skiing speed at identical course locations. Section times and instantaneous speed for each skier was calculated from the GNSS data, which was downloaded from the device internal storage and analysed using the manufacturer provided software (Catapult OpenField; Catapult Sports, Melbourne Australia). The typical error of the GNSS system has been reported to be; Easting: 0.31 ± 0.06 m; Northing: 0.40 ± 0.12 m; Vertical: 0.58 ± 0.15 m (Gløersen et al., 2018b).

### Statistical Analyses

Two-tailed Pearson's correlation coefficients were computed with IBM SPSS statistics 26 (IBM Co., Armonk, New York, USA) to examine the relationships between section times (within each separate lap) and total race time. The instantaneous speed curves (1-dimensional; 1D data) from all course sections that were significantly related to total race time were analysed using a SPM procedure using open-source SPM 1D software (Pataky, 2012) in MATLAB R2018a (The MathWorks, Inc., Natick, Massachusetts, USA). For each section of interest (based on Pearson's correlations), SPM 1D one-tailed linear regression models were applied to investigate the relationships between instantaneous speed and either section time or total race time. This resulted in SPM{t} curves with a critical threshold set at α = 0.05. The SPM{t} curve represents the alpha value of the relationship between instantaneous speed and either section or total race time at every 1-m integer. Where the SPM{t} values exceeded the critical threshold, instantaneous speed was considered to be significantly related to the section or total race time. The course locations where the SPM{t} curve exceeded the critical threshold (i.e., the course locations where instantaneous speed and section time or total race time were significantly related) were computed. These sections are termed “SPM clusters.” For each SPM cluster, the position within the section (start to end), the distance, duration, mean speed and the time difference between the fastest and the slowest skier was computed. Data are presented as mean ± standard deviation.

## Results

### Women

Total race time was 28 min 44 ± 58 s. Section times and their associated correlations with total race time are presented in Table 1. There were significant positive linear relationships between section time and total race time for all sections (*p* ≤ 0.040), except for the three latter downhill sections (S4, S6, and S8) on lap 1 and the second uphill section (S3) on lap 2.

**Table 1:** Mean ± standard deviation section times and Pearson correlation coefficients (r) between section times and total race time, with the associated 95% confidence interval (CI), for the women (*n* = 9).

	**Lap 1**		**Lap 2**	
	**Time (s)**	**r (95% CI)**	**Time (s)**	**r (95% CI)**
S1	226 ± 10	0.690^*^ (0.047 to 0.928)	231 ± 11	0.945^*^ (0.755 to 0.989)
S2	102 ± 2	0.747^*^ (0.164 to 0.943)	106 ± 3	0.939^*^ (0.732 to 0.987)
S3	67 ± 3	0.903^*^ (0.596 to 0.980)	54 ± 3	0.666 (0.003 to 0.922)
S4	36 ± 1	0.288 (−0.465 to 0.799)	51 ± 2	0.879^*^ (0.515 to 0.974)
S5	243 ± 11	0.954^*^ (0.790 to 0.990)	250 ± 11	0.919^*^ (0.655 to 0.983)
S6	55 ± 1	0.666 (0.003 to 0.922)	53 ± 1	0.699^*^ (0.065 to 0.931)
S7	61 ± 3	0.887^*^ (0.542 to 0.976)	57 ± 3	0.824^*^ (0.354 to 0.962)
S8	24 ± 1	0.569 (−0.152 to 0.895)	46 ± 2	0.764^*^ (0.203 to 0.925)
S9	62 ± 3	0.847^*^ (0.419 to 0.967)	-	-

* *p < 0.05*.

**Figure 2:**
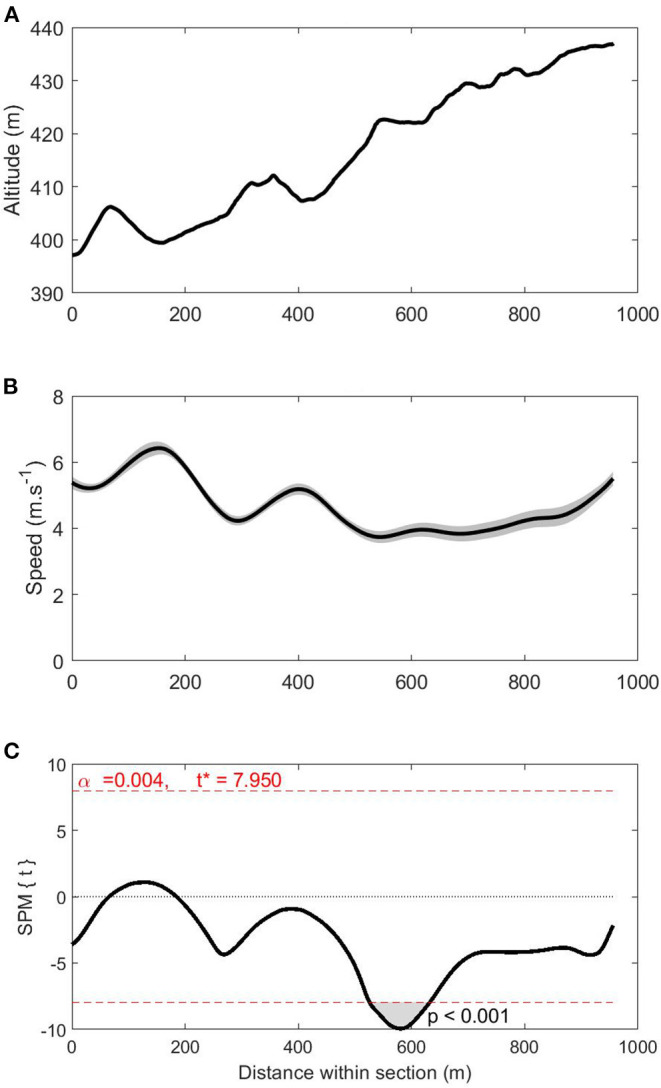
Altitude (panel **A**), speed (panel **B**), and statistical parametric mapping (SPM{t}) curves (panel **C**) for the women in S1 on lap 2 (i.e., the course section where race time was significantly related to instantaneous skiing speed). The shaded area on the speed curve represents the 95% confidence interval. The shaded area on the SPM{t} curve shows the course location where a significant relationship exists between race time and instantaneous speed (at 523–632 m). *p < 0.05.

The SPM regressions revealed that total race time was associated with instantaneous speed during parts of S1 on lap 2 (*t* = 7.950; *p* = 0.004; Figure 2). Specifically, shorter total race times were related to higher speeds over a 109-m cluster (at 523–632 m) within S1 on lap 2 (*p* < 0.001). During this cluster, mean skiing speed was 3.8 ± 0.2 m·s^−1^ and the fastest skier gained 3.9 s on the slowest skier. No other significant associations were identified between total race time and instantaneous speed on specific course sections.

Table 2 displays the SPM clusters within each section where section time was significantly related to total race time. For each of these clusters the mean ± standard deviation time, speed and time gain (i.e., the time difference between the fastest and slowest skier) is also displayed. Despite significant relationships between section times and total race time, SPM regressions did not reveal any clusters where section time or total race time was related to instantaneous speed in S2, S3, S4, S6, or S8. The greatest time gains were observed in clusters on lap 1, in the latter part of the first uphill section (S1) and in the flat section (S9). Within these 2 clusters, the fastest skier gained 6.9 s and 7.3 s, respectively, on the slowest skier. Over all sections on the first and second laps, respectively, the fastest skier gained 17.7 s and 1.8 s on the slowest skier.

**Table 2:** Characteristics of the SPM clusters where section time was significantly related to instantaneous speed for the women, with mean ± standard deviation time, velocity and time gain (i.e., the time difference between the fastest and slowest skier) for each cluster.

**Lap**	**Section**	**Cluster start (m)**	**Cluster end (m)**	**Cluster distance (m)**	**Cluster duration (s)**	**Speed (m·s^**−1**^)**	**Time gain (s)**
1	1	623	644	21	4.7 ± 0.2	4.3 ± 0.2	0.7
1	1	666	821	155	36.4 ± 2.0	4.3 ± 0.2	6.9
1	5	496	527	31	11.0 ± 0.6	2.8 ± 0.2	1.8
1	7	172	204	32	7.4 ± 0.3	4.4 ± 0.2	1.0
1	9	130	436	306	48.4 ± 2.2	6.4 ± 0.6	7.3
2	1	635	665	30	7.7 ± 0.5	3.9 ± 0.2	1.2
2	7	219	239	20	4.3 ± 0.2	4.6 ± 0.2	0.6

### Men

Total race time was 38 min 37 ± 57 s. Section times and their associated correlations with total race time are presented in Table 3. There were significant positive linear relationships between section time and total race time for S5 (i.e., the longest of the four climbs) on all three laps (*p* < 0.05). In addition, there were significant positive linear relationships between section time and total race time for S1 (i.e., the second longest climb) on laps 2 and 3 (*p* < 0.01). There was also a significant positive linear relationship between section time and total race time for S9 (i.e., the flat section) on lap 2 (*r* = 0.649, *p* = 0.042).

**Table 3:** Mean ± standard deviation section times (s) and Pearson correlation coefficients (r) between section times and total race time, with the associated 95% confidence interval (CI), for the men (*n* = 10).

	**Lap 1**		**Lap 2**		**Lap 3**	
	**Time (s)**	**r (95% CI)**	**Time (s)**	**r (95% CI)**	**Time (s)**	**r (95% CI)**
S1	194 ± 5	0.334 (−0.374 to 0.796)	197 ± 9	0.888^*^ (0.587 to 0.973)	204 ± 10	0.784^*^ (0.305 to 0.946)
S2	94 ± 3	0.199 (−0.493 to 0.736)	98 ± 2	0.600 (−0.048 to 0.892)	99 ± 3	0.277 (−0.427 to 0.772)
S3	56 ± 2	0.438 (−0.264 to 0.837)	45 ± 2	0.629 (−0.001 to 0.902)	60 ± 3	0.481 (−0.214 to 0.852)
S4	33 ± 1	−0.098 (−0.685 to 0.567)	48 ± 1	0.597 (−0.052 to 0.892)	35 ± 1	0.280 (−0.424 to 0.772)
S5	210 ± 10	0.758^*^ (0.245 to 0.939)	219 ± 10	0.854^*^ (0.484 to 0.965)	218 ± 12	0.804^*^ (0.352 to 0.962)
S6	51 ± 1	0.504 (−0.184 to 0.860)	51 ± 2	0.496 (−0.195 to 0.858)	50 ± 1	0.306 (−0.401 to 0.784)
S7	52 ± 2	0.381 (−0.327 to 0.815)	55 ± 3	0.485 (−0.208 to 0.854)	49 ± 2	0.079 (−0.579 to 0.675)
S8	23 ± 1	0.220 (−0.475 to 0.746)	24 ± 1	0.360 (−0.349 to 0.807)	43 ± 1	0.407 (−0.299 to 0.825)
S9	55 ± 2	0.524 (−0.158 to 0.867)	57 ± 2	0.649^*^ (0.033 to 0.908)	-	-

* *p < 0.05*.

**Figure 3:**
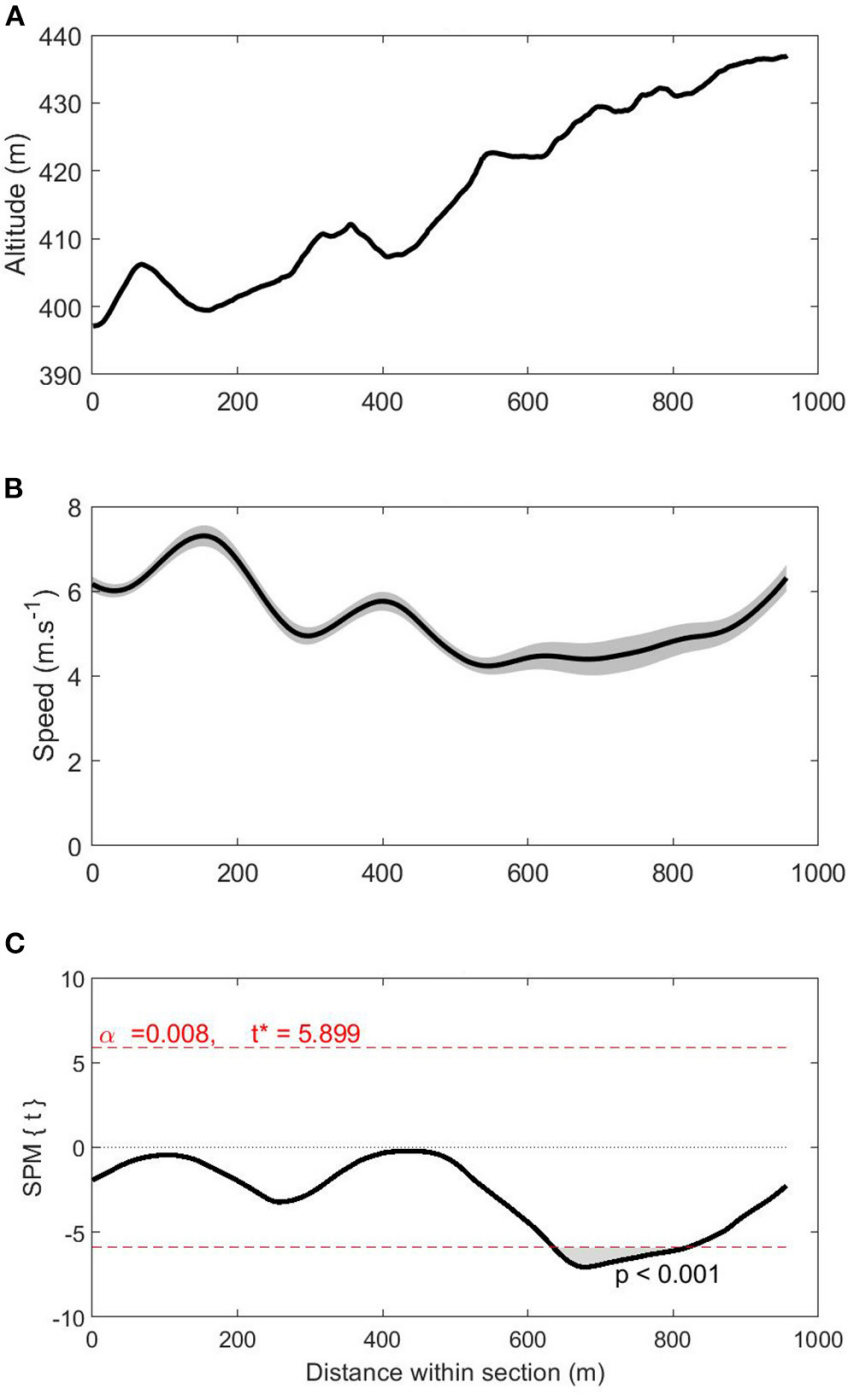
Altitude (panel **A**), speed (panel **B**), and statistical parametric mapping (SPM{t}) curves (panel **C**) for the men in S1 on lap 2 (i.e., the course section where race time was significantly related to instantaneous skiing speed). The shaded area on the speed curve represents the 95% confidence interval. The shaded area on the SPM{t} curve shows the course location where a significant relationship exists between race time and instantaneous speed (at 635–819 m). *p < 0.05.

The SPM regressions revealed that total race time was associated with instantaneous speed during parts of S1 on lap 2 (*t* = 5.899; *p* = 0.008; Figure 3). Specifically, shorter race times were related to higher speeds over a 184-m cluster (at 635–819 m) within S1 on lap 2 (*p* < 0.01). During this cluster, mean skiing speed was 4.6 ± 0.4 m·s^−1^ and the fastest skier gained 10.6 s on the slowest skier. No other significant associations were identified between total race time and instantaneous speed on specific course sections.

Table 4 displays the SPM clusters within each section where section time was significantly related to total race time. For each of these clusters the mean ± standard deviation time, speed and time gain (i.e., the time difference between the fastest and slowest skier) is also displayed. The greatest time gains were observed in S5 on the second lap, where the fastest skier gained 20.4 s on the slowest skier. Over all clusters in S5 for all three laps combined, the fastest skier gained 51.7 s on the slowest skier. Over all sections on the first, second and third laps, respectively, the fastest skier gained 15.3 s, 35.5 s, and 26.6 s on the slowest skier.

**Table 4:** Characteristics of the SPM clusters where section time was significantly related to instantaneous speed for the men, with mean ± standard deviation time, velocity, and time gain (i.e., the time difference between the fastest and slowest skier) for each cluster.

**Lap**	**Section**	**Cluster Start (m)**	**Cluster End (m)**	**Cluster Distance (m)**	**Cluster duration (s)**	**Speed (m·s^**−1**^)**	**Time gain (s)**
1	5	187	354	167	41.6 ± 2.0	4.0 ± 0.2	5.9
1	5	433	617	184	54.1 ± 3.4	3.4 ± 0.4	9.4
2	1	557	741	184	41.9 ± 3.2	3.9 ± 0.6	8.7
2	5	268	647	379	108.3 ± 6.7	3.6 ± 0.4	20.4
2	9	154	436	282	41.5 ± 2.2	6.8 ± 0.5	6.4
3	1	253	312	59	12.2 ± 0.5	4.9 ± 0.3	1.6
3	1	479	699	220	51.2 ± 3.1	4.3 ± 0.3	9.0
3	5	163	277	114	29.7 ± 1.7	3.8 ± 0.2	5.1
3	5	372	454	82	24.7 ± 1.7	3.4 ± 0.3	5.0
3	5	496	592	96	28.5 ± 2.1	3.4 ± 0.3	5.9

## Discussion

This is the first study to our knowledge that has analysed micro-pacing strategies during a freestyle distance XC skiing race, using a method that allows comparison of skiing speed at standardised course locations. The main findings were that: (1) skiers with shorter total race or section times skied with higher instantaneous speeds in specific uphill and flat sections of the course; (2) compared to the less successful women, the more successful women skied faster in clusters within the three longer uphill sections (i.e., in S1 and S7 on both laps and in S5 on lap 1) and in the flat section (i.e., S9 on lap 1); 3) the more successful men skied faster in clusters within the two longest uphill sections (i.e., S1 on laps 2 and 3 and S5 on all 3 laps) and in the flat section (S9) on lap 2; 4) the fastest woman gained the most time in relation to the slowest woman in S1 and S9 on lap 1, while the fastest man gained the most time in relation to the slowest man in S5 on lap 2; 5) statistical parametric mapping is a valuable tool for analysing micro-pacing strategies and could be practically useful when providing pacing and performance feedback to athletes and coaches.

Previous research has identified the importance of uphill sections in determining overall performance in XC skiing (Andersson et al., 2010; Sandbakk et al., 2011, 2016). Specifically, Sundström et al. (2013) used computerised modelling to demonstrate that an optimal pacing strategy is characterised by increased propulsive power during uphill course sections. In addition, Ihalainen et al. (2020) observed that instantaneous skiing speeds in particular parts of uphill sections were related to section or total race times in a women's classic sprint XC skiing race. The present study support and extend these previous findings by describing the micro-pacing strategies during a freestyle distance race for both women and men. In particular, the two longest uphill sections (S1 and S5), as well as the flat course section (S9), contained clusters where section and/or total race times were related to instantaneous skiing speed for both sexes. For example, in the women's race the fastest skier gained 6.9 s on the slowest skier during a cluster in S1 on the first lap, and gained 7.3 s during a cluster in the flat section (S9). In the men's race, the fastest skier gained 51.7 s on the slowest skier over all clusters in S5 for all three laps combined. This finding further contributes to the notion that uphill sections are particularly critical to success in XC skiing and provides valuable information to coaches and skiers to inform pacing strategies and training programs.

The largest time gain between the fastest and slowest female skier in a single course section (i.e., 7.3 s) was observed in the flat section (S9) at the end of the first lap. By contrast, the largest time gain observed between the fastest and slowest male skier (i.e., 20.4 s) was in the longest uphill section (S5) on the second lap. These findings might reflect different micro-pacing strategies adopted between sexes, as well as different relative strengths in women and men. Sex differences in the fastest vs. slowest skier might be explained by variations in skiing speed during transitional periods between flat and uphill sections (Ardigò et al., 2020). Although the FIS points were similar between sexes in the present study, there was a greater variation in the male athletes, which might explain why larger time differences were observed in this cohort. Additionally, sex differences in the fastest vs. slowest skier might be explained by variations in relative power outputs (Andersson et al., 2019). Further, Losnegard et al. (2016) observed that the most successful male skiers were able to maintain a more even lap-to-lap pacing strategy in comparison to their slower counterparts during a distance XC skiing race. On the other hand, there was little difference in the lap-to-lap pacing strategy between the fastest and slowest female skiers (Losnegard et al., 2016). The present study supports these findings, whereby the largest time difference between the fastest and slowest skiers was observed on the first lap for the women, but on the second and third laps for the men. This suggests that lower-performing male skiers might benefit from adopting a more even lap-to-lap pacing strategy. Further studies could attempt to investigate the effects of altering pacing strategies in the field, and/or implementing training strategies to improve pacing among competitive XC skiers.

The present study demonstrated that shorter total race times were not related to higher instantaneous skiing speeds during downhill sections for either the women or men. However, section time in S2 (one of the downhill sections) was related to total race time for the women. Previous research has reported that downhill section times were not related to shorter race times in XC sprint skiing races (Andersson et al., 2010; Sandbakk et al., 2011). However, these studies only observed pacing strategies for men participating in sprint competitions, but not for women. The results from the present study suggest that downhill skiing performance might be more important for success among women during freestyle distance competitions.

Previous research has mainly focused on lap-to-lap pacing strategies in XC skiing (Andersson et al., 2010; Sandbakk et al., 2011, 2016; Swarén and Eriksson, 2019). For example, lap times in XC ski races tend to become slower over the duration of a race (i.e., positive pacing) (Stöggl et al., 2018). Whilst this information is useful, it provides limited understanding of a skier's distribution of effort within each lap. The present study provides novel insights for XC skiers and coaches and demonstrates that SPM analyses can provide practical information to optimise pacing strategies. Using historical data, coaches and sports scientists could use SPM analyses to identify crucial components for success on various courses and race formats in order to guide pacing strategies prior to an event. In addition, individual skiers' strengths and weaknesses can be identified and used to guide training programs.

A limitation of the present study is the lack of accounting for skiers using drafting tactics. The methodological approach assumed that skiers adopted micro-pacing strategies independent of other competitors on the course. Although the race was performed as an individual time-trial, skiers were still able to overtake and be overtaken, which may have led to drafting tactics having positive aerodynamic and physiological effects (Bilodeau et al., 1994, 1995). Accordingly, it is possible that individual micro-pacing strategies were influenced by other skiers on the course. Second, waxing strategies of the skis can affect overall race performance. Because ski waxes have different effects at different skiing speeds, waxing strategies might influence section times along the course and hence the SPM analyses. Third, the data in the present study represent a freestyle XC skiing distance race from only one course location. The generalisability of these findings to other courses with different topographical profiles, race distances, techniques, and skiers remain to be confirmed. Nevertheless, the methodological approach in this study has utility for sports scientists and coaches to optimise pacing strategies and improve performance for any skier at any course location.

## Conclusion

Specific uphill and flat course sections were identified where skiers with shorter total race times skied with faster instantaneous speeds compared to skiers with longer race times. More specifically, successful skiers had faster instantaneous speeds in some clusters of the uphill sections, as well as on the flat section of the course. In addition, this study identified that relative micro-pacing strategies differed between the women and men during their freestyle distance XC skiing races. Specifically, in the women's race the largest time gain between the fastest and slowest skier was observed in the flat section at the end of the first lap (S9; 7.3 s). By contrast, the largest time gain observed between the fastest and slowest male skier was in the longest uphill section on the second lap (S5; 20.4 s). Finally, SPM analyses can be used by coaches and athletes to identify individual athletes' strengths and weaknesses for guiding training programs and to optimise pacing strategies.

## Data Availability Statement

The raw data supporting the conclusions of this article will be made available by the authors, without undue reservation.

## Ethics Statement

The studies involving human participants were reviewed and approved by the regional ethical review board of Umeå University, Sweden (#2018-441-32M). The patients/participants provided their written informed consent to participate in this study.

## Author Contributions

CS performed the data analysis, statistical analyses, and wrote the manuscript. SC performed the SPM analysis, constructed SPM figures and provided editorial assistance in writing the manuscript. ØK provided editorial assistance in writing the manuscript. MS assisted with data analysis and provided editorial assistance in writing the manuscript. SI participated in the design of the study and collected the data. KM participated in the design of the manuscript, supervised the project and provided editorial assistance in writing the manuscript. All authors have read and approved the final version of the manuscript.

## Conflict of Interest

The authors declare that the research was conducted in the absence of any commercial or financial relationships that could be construed as a potential conflict of interest.

## Publisher's Note

All claims expressed in this article are solely those of the authors and do not necessarily represent those of their affiliated organizations, or those of the publisher, the editors and the reviewers. Any product that may be evaluated in this article, or claim that may be made by its manufacturer, is not guaranteed or endorsed by the publisher.
